# Supplementary material for: Optimizing energy efficiency in heterogeneous networks: An integrated stochastic geometry approach with novel sleep mode strategies and QoS framework
Source: PLoS One. 2024 Feb 26;19(2):e0296392. doi: 10.1371/journal.pone.0296392 (PMC10896529; doi:10.1371/journal.pone.0296392)
Supplement: Attachment — Submitted filename: Response to the reviewer.docx [file pone.0296392.s001.docx]

**Response to the reviewers**

We thanks the reviewers for their critical assessment of our research work. In following, we address their concerns point by point.

**Reviewer 1:**

1. **In abstract, the value achieved in improving energy consumption and its difference from the most efficient method must be mentioned.**

**Response:**

The abstract have been updated to include the achieved value in improving energy consumption and highlight the difference from the most efficient method.

2. **In abstract, the research methodology is not well articulated and is somewhat messy.**

**Response:**

The abstract is revised to provide a clearer and more organized articulation of the research methodology.

3. **In the introduction, Figure 1 does not clearly list the purpose of its existence in terms of energy efficiency over the recent years.**

In the introduction, the details about Figure 1 have been revised with increased clarity, accompanied by an explanation of its existence concerning energy efficiency over recent years.

4. **In line 70, the fundamental challenges are not mentioned**

**Response:**

Line 70 is being revised and a detailed discussion regarding the fundamental challenges is included in the context of the study.

5. **In the line 83, it is better to delete a (random)**

**Response**

(random) has been deleted

6. **In line 99, what are the three approaches, it should be listed and explain what it is HetNets in line 100.**

**Response:**

line 99 and 100, are modified to explicitly list the three approaches along with the explanation of HetNets to enhance clarity and understanding.

7. **In related works, due to the importance of both DAS and LAS, its working mechanism must be explained.**

**Response:**

In the related works section, the working mechanisms of both DAS and LAS are explained in detail to highlight their importance.

8. **It is preferable to divide the related work according to common mechanisms.**

**Response:**

Related work has been revised according to common mechanism.

9. **The “Related Work” and “Introduction” sections lack of enough references. I strongly recommend that the author improve this section by adding references that support all the claims, the challenges of QoS, and motivation of the problem. The author may precisely and comprehensively point out the current issues and existing solutions.**

**Response:**

The "Related Work" and "Introduction" sections are enhanced by incorporating additional references to substantiate claims, address QoS challenges, and provide motivation for the problem. We have ensured a thorough and detailed discussion of present challenges and the solutions that currently exist.

10. **In line 169, please replace oversimplified ◊ simplified**

**Response:**

In line 169, the term "oversimplified" is replaced with "simplified" for more accurate representation.

11. **In lines 174-175, “EE algorithms based on sleep mode strategies, the negative impact on QoS (174 when BSs are turned 175 into sleep mode) has been ignored in various research the publications”, What is the proof for this claim?**

**Response:**

The proof for the claim regarding the neglect of the negative impact on QoS when base stations are turned into sleep mode in EE algorithms based on sleep mode strategies (lines 174-175) is provided through references to relevant research publications [1-3].

12. **Contributions are worded superficially and do not fit with the proposed work. They must be explained in depth**

**Response:**

The contributions are revised and explained in greater depth to align more closely with the proposed work.

13. **Section 6 . Mathematical Preliminaries, Lacks references**

**Response:**

In Section 6, "Mathematical Preliminaries," references are added to provide proper citation and support for the mathematical concepts presented.

14. **The role of stochastic geometry in the work is not entirely clear**

**Response:**

The role of stochastic geometry in the work is clarified to ensure a better understanding of its significance and application in the context of the research work.

15. **In line 237, “that the density of the points will remains constant throughout the Euclidean space.”, the constant of these points may affect accuracy**

**Response:**

Maintaining a constant density of points throughout Euclidean space in stochastic geometry models, despite potential variations in this constant, does not significantly affect the accuracy of the analysis. The decision to keep the point density constant is often driven by the need to establish a standardized and consistent basis for comparative analysis, enabling researchers to evaluate Quality of Service (QoS) parameters in a fair and comparable manner across different studies[4-7].

The decision to maintain a constant point density in stochastic geometry models is a deliberate choice to facilitate fair and comparable analyses. While it may not perfectly mirror real-world variability, the relative comparisons and insights derived from this standardized approach contribute valuable knowledge to the field, allowing researchers to build upon and extend existing literature in a meaningful way.

16. **In section 7. System model, There is a bit of ambiguity and messy as to whether the proposed work is two-tier or multi-tier**

**Response:**

The ambiguity and confusion in Section 7 regarding whether the proposed work is a two-tier or multi-tier system model are addressed and clarified to ensure a clear and coherent presentation of the system architecture.

17. **In line 267, these conditions were not mentioned or clarified**

**Response:**

The specific conditions were not explicitly detailed, they are derived from a referenced source[8].

18. **In lines 284-285, this wake-up time requires clarification**

**Response:**

The wake-up time mentioned in lines 284-285 are clarified to provide a more detailed explanation and enhance understanding.

19. **Table 3, how were these values assigned?**

**Response:**

The wake-up time values in the research were obtained from [9], which includes the impact of various wake-up times on the utilization of sleep modes, particularly in the context of small BSs. The values presented in the Table3 for small cell BSs took into account factors such as network conditions, traffic load, and specific power-saving modes, contributing to an analysis of the variability in wake-up times.

20. **In lin2 309, the percentages are 67% and 98%, how were they obtained, and the context of the work is here**

**Response:**

The percentages of 67% and 98% were derived from our simulated results, and a detailed discussion of the methodology and findings can be found in the results section of our work. In this section, we provide a comprehensive analysis of the performance metrics, and simulations, offering insights into how the proposed D-DOSS outperforms existing techniques and achieves notable improvements in Energy Efficiency. The specific steps taken to calculate these percentages, as well as the contextual details surrounding our simulation methodology, are thoroughly outlined to for deeper understanding of our research outcomes.

21. **The variables and factors of Equation 13 and 16 are not explained**

**Response:**

The variables and factors in Equations 13 and 16 are being explained and updated to provide a clear understanding of their significance and roles within the context of the study.

22. **In line 22, the transmission power was assumed to be constant, which may affect the validity of the results of the proposed work**

**Response:**

The assumption of constant transmission power in the proposed work was made for simplicity, facilitating a clear analysis of the impact of sleep modes on energy efficiency. This aligns with the common practice in related studies, allowing for meaningful comparisons. By keeping transmission power constant, the research can specifically focus on the isolated effects of sleep mode variations without the complexity introduced by changes in power levels. While this assumption simplifies the models and simulations, it acknowledges the need for a manageable and focused investigation into the targeted impact of sleep modes on energy consumption, providing valuable insights within a practical context.

23. **The algorithms are good in terms of formulation, but require more clarification to explain the role of each one were these values established?**

**Response:**

We appreciate the reviewer's acknowledgment of the algorithms' formulation. While we understand the importance of clarity, we intentionally designed the algorithms to be concise and straightforward. The specific values and parameters have been established based on standard practices and considerations specific to our study. Unfortunately, we are unable to delve into further details, as the algorithms are presented in a manner that aligns with our study's objectives. If there are specific concerns or questions about the overall outcomes or results, we are open to discussing those aspects more broadly.

**Reviewer #2:**

1. **It is possible to add references to Table 1 according to the mentioned distribution**

**Response:**

Table 1 has been updated with the references according to the mentioned distribution

2. **The research papers in Related Work are not arranged according to year of publication (from oldest to newest). In addition to the possibility of mentioning the failures of each research.**

**Response:**

We appreciate the reviewer's feedback on the organization of the **Related Work** section. We deliberately structured the section based on the level of complexity in the proposed strategies rather than the chronological order of publication. For clarity and a coherent narrative, we introduced the RSM strategy first, followed by LAS and DAS, which represent more advanced techniques. Changing the order based on publication year could disrupt the logical progression of complexity and may lead to a less intuitive presentation. However, we have ensured that each research paper's contributions and limitations, including potential shortcomings, are thoroughly discussed to provide a comprehensive understanding for readers.

3. **It is possible to discuss the results and build clear and scientific conclusions, in addition to setting a future outlook for researchers in this field**

**Response:**

Thank you for your input. To foster a comprehensive comprehension, the discussion of results is being conducted within the results section. Furthermore, we have incorporated limitations and future recommendations to enhance the depth and clarity of understanding for researchers in this domain.

4. **Standardize the format of references**

**Response:**

References have been updated and formatted according to the IEEE 2006 standard.

**References:**

[1]	C. K. Agubor, A. O. Akande, C. R. J. I. W. Opara, and M. Technologies, "On-off Switching and Sleep-mode Energy Management Techniques in 5G Mobile Wireless Communications–A Review," vol. 6, pp. 40-47, 2022.

[2]	I. Ashraf, F. Boccardi, and L. Ho, "Sleep mode techniques for small cell deployments," *IEEE Communications Magazine,* vol. 49, no. 8, 2011.

[3]	J. Wu, Y. Zhang, M. Zukerman, and E. K.-N. Yung, "Energy-efficient base-stations sleep-mode techniques in green cellular networks: A survey," *IEEE communications surveys & tutorials,* vol. 17, no. 2, pp. 803-826, 2015.

[4]	J. G. Andrews, F. Baccelli, and R. K. Ganti, "A tractable approach to coverage and rate in cellular networks," *IEEE Transactions on Communications,* vol. 59, no. 11, pp. 3122-3134, 2011.

[5]	J. G. Andrews, S. Singh, Q. Ye, X. Lin, and H. S. Dhillon, "An overview of load balancing in HetNets: Old myths and open problems," *IEEE Wireless Communications,* vol. 21, no. 2, pp. 18-25, 2014.

[6]	H. S. Dhillon, R. K. Ganti, and J. G. Andrews, "Load-aware modeling and analysis of heterogeneous cellular networks," *IEEE Transactions on Wireless Communications,* vol. 12, no. 4, pp. 1666-1677, 2013.

[7]	A. Ghosh *et al.*, "Heterogeneous Cellular Networks: From Theory to Practice," *IEEE Communications Magazine,* p. 55, 2012.

[8]	I. Ashraf, F. Boccardi, and L. Ho, "Power savings in small cell deployments via sleep mode techniques," in *Personal, indoor and mobile radio communications workshops (PIMRC Workshops), 2010 IEEE 21st international symposium on*, 2010, pp. 307-311: IEEE.

[9]	W. Vereecken *et al.*, "The effect of variable wake up time on the utilization of sleep modes in femtocell mobile access networks," in *Wireless On-demand Network Systems and Services (WONS), 2012 9th Annual Conference on*, 2012, pp. 63-66: IEEE.
